# Malondialdehyde as an independent predictor of body mass index in adolescent girls

**DOI:** 10.5937/jomb0-39044

**Published:** 2023-03-15

**Authors:** Aleksandra Klisic, Maja Malenica, Jelena Kostadinovic, Gordana Kocic, Ana Ninic

**Affiliations:** 1 University of Montenegro, Faculty of Medicine, Primary Health Care Center, Podgorica, Montenegro; 2 University of Sarajevo, Faculty of Pharmacy, Department of Pharmaceutical Biochemistry and Laboratory Diagnostics, Sarajevo, Bosnia and Herzegovina; 3 Clinical Hospital Center Bezanijska kosa, Belgrade; 4 University of Nis, School of Medicine, Department of Medical Biochemistry, Nis; 5 University of Belgrade, Faculty of Pharmacy, Department for Medical Biochemistry, Belgrade

**Keywords:** adolescents, inflammation, obesity, oxidative stress, adolescenti, inflamacija, gojaznost, oksidativni stres

## Abstract

**Background:**

Given the fact that the studies that examined oxidative stress in relation to obesity that included late adolescents are scarce and show inconclusive results we aimed to investigate a wide spectrum of nitro-oxidative stress biomarkers i.e., malondialdehyde (MDA), xanthine oxidase (XO), xanthine oxidoreductase (XOD), xanthine dehydrogenase (XDH), advanced oxidation protein products (AOPP) and nitric oxide products (NOx), as well as an antioxidative enzyme, i.e., catalase (CAT) in relation with obesity in the cohort of adolescent girls ages between 16 and 19 years old.

**Methods:**

A total of 59 teenage girls were included in this cross-sectional study. Binary logistic regression analysis was performed to examine possible associations between biochemical and nitro-oxidative stress markers and body mass index (BMI).

**Results:**

There were not significant differences between oxidative stress markers between normal weight and overweight/obese girls (i.e., AOPP, XOD, XO, XDH) and CAT, except for MDA (p<0.001) and NOx (p=0.010) concentrations which were significantly higher in overweight/obese adolescent girls. Positive associations were evident between BMI and high sensitivity C-reactive protein (hsCRP) (OR=2.495), BMI and uric acid (OR=1.024) and BMI and MDA (OR=1.062). Multivariable binary regression analysis demonstrated significant independent associations of BMI and hsCRP (OR=2.150) and BMI and MDA (OR=1.105). Even 76.3% of the variation in BMI could be explained with this Model.

**Conclusions:**

Inflammation (as measured with hsCRP) and oxidative stress (as determined with MDA) independently correlated with BMI in teenage girls.

## Abstract (sr)

**Uvod:**

Imajući u vidu činjenicu da je malo studija koje su ispitivale povezanost oksidativnog stresa i gojaznosti kod adolescenata i da iste pokazuju oprečne rezultate, cilj istraživanja je bio da se ispita povezanost širokog spektra biomarkera nitro-oksidativnog stresa tj. malondialdehida (MDA), ksantin oksidaze (XO), ksantin oksidoreduktaze (XOD), ksantin dehidrogenaze (XDH), produkata uznapredovale oksidacije proteina (AOPP) i produkata azot-monoksida (NOx), kao i enzima antioksidativne zaštite, tj. katalaze (CAT) i gojaznosti u kohorti adolescentkinja starosne dobi izme|u 16 i 19 godina.

**Metode:**

Ukupno 59 tinejdžerki je uključeno u ovu studiju preseka. Binarna logistička regresija je primenjena u cilju ispitivanja potencijalne povezanosti između biohemijskih markera i markera nitro-oksidativnog stresa i indeksa telesne mase (ITM).

**Rezultati:**

Nije uočena razlika u biomarkerima oksidativnog stresa između normalno uhranjenih i predgojaznih/gojaznih adolescentkinja (odnosno AOPP, XOD, XO, XDH) i CAT, osim u vrednostima MDA (p<0,001) i NOx (p=0,010) koje su bile značajno veće kod predgojaznih/gojaznih adolescentkinja. Pozitivna korelacija je utvrđena izme|u ITM-a i visokosenzitivnog C-reaktivnog proteina (hsCRP) (OR=2,495), ITM-a i mokraćne kiseline (OR=1,024) i ITM-a i MDA (OR=1,062). Multivarijanta binarna regresija je pokazala nezavisnu povezanost ITM-a i hsCRP (OR=2,150), kao i ITM-a i MDA (OR=1,105). Čak 76,3% varijabiliteta ITM-a može biti objašnjeno ovim modelom.

**Zaključak:**

Inflamacija (merena hsCRP-om) i oksidativni stres (meren malondialdehidom) nezavisno koreliraju sa ITM kod adolescentkinja.

## Introduction

The prevalence of obesity has reached high prevalence both in young and adult populations. Recent reports indicate that the prevalence of obesity in women has increased 2.5-fold (i.e., from 6% to 15%) in the last 40 years [1]. Also, a higher percentage of body fat was shown in women compared to men, which indicates that pathophysiological processes that underly obese state, such as in flammation and oxidative stress may have a stronger impact on obesity related disorders in women compared to men [2].

Obesity in adolescents often tracks into the adult period and is regarded as one of the most serious public health concerns nowadays. Almost 17% of children are with obesity in the USA, whereas one out of three children is overweight/obese in Europe. Similar results are shown in Africa, where obesity in female adolescents (36.1%) is more prevalent than in males [3] [4].

These metabolic changes are mainly attributed to sedentary lifestyle and unhealthy dietary pattern (e.g., easy access to fast-food and sugar-sweetened beverages) [5].

Obesity leads to many metabolic disturbances, such as polycystic ovary syndrome (PCOS), fatty liver disease, diabetes mellitus type 2 and cardiovascular disease [6] [7] [8] [9] [10].

Oxidative stress and inflammation are the main pathophysiological features of the obesity-related disorders [5] [10]. The over production of reactive nitrogen or oxygen species (RNS/ROS) (i.e., prooxidants) over the antioxidant defence systems (i.e., antioxidants) results in oxidative stress. To date, the precise mechanism by which prooxidants and antioxidants influence metabolic disturbances are not fully enlightened [5].

Despite the large number of studies that explored different oxidative stress biomarkers, the results are still inconclusive and the biomarker that best reflects oxidative stress in obesity has not been found yet, given the fact that RNS/ROS are not easily measured due to their short half-life and their low levels [10] [11].

Therefore, the determination of lipid and protein oxidation products, as well as by-products of DNA modification take their place in clinical settings [11].

Malondialdehyde (MDA) is among the most common used biomarker that reflects lipid peroxidation. The key targets of this process are polyunsaturated fatty acids, mostly arachidonic and linoleic acid. During the reaction of these molecules with ROS the autocatalytic reaction of lipid peroxidation occurs, with consequent formation of secondary by-products, such as MDA, isoprostanes and trans-4-hydroxy-2-nonenal [11].

The commonly used biomarkers of protein oxidative modification are advanced oxidation protein products (AOPP) [11].

Nitric oxide (NO) is one of the biomarkers of nitro-oxidative stress and the major determinant of vascular tone and energy metabolism [12]. NO also favors oxidative stress by regulating lipid peroxidation and favors the generation of MDA [13].

Xanthine oxidase (XO) represents an oxidant form of the enzyme xanthine oxidoreductase (XOD). XO is the main culprit for liberation of ROS in circulation. XOD is responsible for the conversion of purine bases to uric acid and is presented as xanthine dehydrogenase (XDH) under physiological conditions [14]. In obesity-metabolic disorders, when antioxidative defense enzymes are depleted [such as superoxide dismutase (SOD), catalase (CAT), glutathione peroxidase (GPx)] [11], XOD is being converted into XO [14].

Given the fact that the prevalence of overweight/obesity among teenage girls is inconsistently reported due to the different ages of adolescents included in the research [15] and since the studies that examined oxidative stress in late adolescents are scarce and show inconclusive results we aimed to investigate a wide spectrum of nitro-oxidative stress biomarkers [i.e., MDA, AOPP, XOD, XO, XDH) and nitric oxide products (NOx=nitrates and nitrites), as well as antioxidative enzyme-CAT] in relation with obesity in the cohort of adolescent girls ages between 16 and 19 years old.

## Materials and methods

### Study population

This case-control cross-sectional study included 59 adolescent girls who were reluctant to participate in the research. After obtaining the approval of the Institutional Ethics Committee, girls between the ages 16-19 years (i.e. from the last two grades of the secondary schools in Podgorica, Montenegro) were consecutively included. Each girl provided written informed consent. The parental informed consent was also provided for girls younger than 18 years. The exclusion criteria were any sign/symptom of inflammatory disease, thyroid diseases, diabetes, autoimmune diseases, any medication use, cigarette smoking, alcohol consumption, pregnancy, irregular menstrual cycle. Also, girls who did not keep their body weight stable in the last three months were excluded from the study, as well as girls that exhibited high sensitivity C-reactive protein (hsCRP) 10 mg/L to minimize the confounding factors and sources of inflammation and oxidative stress other than obesity.

### Biochemical analyses

Blood sampling was performed after an overnight fast of at least 8 hours, between 7:00 h and 10:00 h, a.m. the same morning when anthropometric measurements were obtained.

The samples were provided in the tube with a serum separator and clot activator and after clotting within 30 minutes, the samples were centrifuged for 10 minutes at 3000xg (at room temperature). Serum levels of triglycerides (TG), high-density lipoprotein cholesterol (HDL-c), low-density lipoprotein cholesterol (LDL-c), total cholesterol (TC), creatinine, uric acid, glucose and hsCRP were measured immediately after the centrifugation of samples on the biochemistry analyzer Roche Cobas c501 (Roche Diagnostics GmbH, Mannheim, Germany). The aliquots of sera were frozen at -80°C until the analyses of the oxidative stress biomarkers.

The oxidative stress parameters were measured as previously described [8] [14] [16]. Briefly, serum AOPP levels were measured by reaction with potassium iodide and glacial acetic acid method [17]. The measurement of serum XOD and XO was related to the liberation of uric acid, whereas xanthine was used as a substrate in the presence of NADH (for XOD) or the absence of NADH (for XO) when molecular oxygen was the electron acceptor [18]. The XDH activity was calculated as follows: XOD - XO activity. Serum NO production is determined after the reduction of nitrate (NO_3_^-^) into nitrite (NO_2_^-^) with cadmium. The measurement of nitrates and nitrites (NO_3_^- ^+ NO_2_), commonly named as NOx, is used as an indicator of NO production [19]. Serum MDA levels (i.e., an end degradation product of lipid hydroperoxides) were determined as a thiobarbituric acid reactive substance [20]. CAT test is related to the breaking down of oxygen from harmful hydrogen peroxide (H_2_O_2_), based on the formation of its complex with ammonium molybdate [21].

### Anthropometric measurements

Anthropometric measurements were obtained as described elsewhere [22]. Body mass index (BMI) was calculated as body weight (kg) divided by body height in meters squared (kg/m^2^). Girls with BMI <25 kg/m^2^ were regarded to be normal weight, whereas girls with BMI 25 kg/m^2^ were regarded as overweight/obese.

### Statistical analysis

Statistical analysis was conducted using SPSS for Windows version 21.0 (SPSS Inc., Chicago, IL, USA). The normality of data distribution was evaluated using the Shapiro Wilk test. Values are expressed as mean ± standard deviation for normally distributed data and median (interquartile range) for data with skewed distribution. Differences among groups were assessed by Student *t*-test and Mann-Whitney *U* test depending on data distribution. Correlations between variables were analysed by calculating Spearman's correlation coefficients (ρ). Binary logistic regression analysis was performed to examine possible associations between biochemical and oxidative stress markers and BMI. Biochemical markers with significant Spearman's correlation coefficients were applied in a multivariable binary regression model to search for possible independent association with BMI. Data from these analyses were given as Odds Ratio (OR) and 95% Confidence interval (CI). The explained variation in BMI was given by Nagelkerke R^2^ value. A *P* value less than 0.05 was considered significant.

## Results

The anthropometric and biochemical values of the study participants according to their BMI, are shown in Table 1. The groups were similar ages and height. There were no differences in glucose, TC, LDL-c, TG levels and creatinine. Nevertheless, HDL-c was significantly lower in overweight/obese, while hsCRP and uric acid levels were significantly higher compared to normal weight adolescent girls.

**Table 1:** Laboratory data in adolescent girls according to BMI. Data are presented as mean ± standard deviation. *P* for Student’s *t*-test.<br>*Data are given as median (interquartile range). *P* for Mann-Whitney *U* test

	BMI ≥ 25 kg/m^2^ Normal weight	BMI ≥ 25 kg/m^2^ Overweight/obese	*P*
Adolescents No.	30	29	
Age, years*	18 (17919)	18 (16–19)	0.287
Weight, kg*	59 (55-65)	75 (71.5-89)	< 0.001
Height, cm*	167.0 (165.0-171.0)	167.0 (165.5–169.5)	0.303
Glucose, mmol/L	4.94±0.36	5.07±0.58	0.463
TC, mmol/L	4.21±0.76	4.34±0.43	0.374
HDL-c, mmol/L	1.66±0.32	1.38±0.41	0.021
LDL-c, mmol/L	2.16±0.61	2.47±0.39	0.056
TG, mmol/L*	0.81 (0.66–0.98)	0.87 (0.73–1.32)	0.217
Creatinine, μmol/L	57±6	58±6	0.791
hsCRP, mg/L*	0.30 (0.30–0.53)	2.00 (0.33–3.70)	0.001
Uric acid, μmol/L	209±37	256±50	0.001

In Table 2 we presented oxidative stress and antioxidative defense markers in examined girls. There were no significant differences between these markers in tested groups except for MDA and NOx concentrations which were significantly higher in overweight/obese adolescent girls.

**Table 2:** Oxidative stress markers in adolescent girls accordingto BMI. Data are presented as mean ± standard deviation. *P* for Student’s *t*-test.<br>*Data are given as median (interquartile range). *P* for Mann-Whitney *U* test

	BMI <25 kg/m^2^	BMI ≥25 kg/m^2^	*P*
MDA, μmol/L*	46.7 (42.4–71.9)	94.6 (74.1–103.5)	<0.001
AOPP, T/L	141.1±34.2	153.5±23.5	0.130
CAT, U/L	60.1±29.4	57.8±26.6	0.649
XOD, U/L	285±66.5	289±57.6	0.941
XO, U/L	144.0±47.2	173.8±52.1	0.114
XDH, U/L*	118 (100–189)	105 (99–116)	0.085
NOx*, μmol/L	51.1 (36.7–68.2)	72.9 (64.7–80.8)	0.010

Further, we conducted correlation analyses for all tested markers and BMI in all participants. We found that BMI was positively related to weight, LDLc, hsCRP, uric acid, MDA and AOPP (Table 3).

**Table 3:** Correlation coefficients of BMI with tested markers in adolescent girls

Variable	ρ	*P*
Age, years	-0.084	0.557
Weight, kg	0.915	<0.001
Height, cm	-0.159	0.293
Glucose, mmol/L	0.141	0.348
TC, mmol/L	0.123	0.414
HDL-c, mmol/L	-0.289	0.051
LDL-c, mmol/L	0.301	0.042
TG, mmol/L	0.213	0.155
Creatinine, μmol/L	0.014	0.926
hsCRP, mg/L	0.485	0.001
Uric acid, μmol/L	0.458	0.001
MDA, μmol/L	0.499	<0.001
AOPP, T/L	0.310	0.036
CAT, U/L	-0.071	0.651
XOD, U/L	-0.073	0.631
XO, U/L	0.019	0.899
XDH, U/L	-0.176	0.248
NOx, μmol/L	0.280	0.063

We performed binary regression analysis to determine in-depth associations of BMI and other markers with a significant Spearman's correlation coefficient (Table 4). Positive associations were evident between BMI and hsCRP (OR=2.495), BMI and uric acid (OR=1.024) and BMI and MDA (OR=1.062). Multivariable binary regression analysis demonstrated significant independent associations of BMI and hsCRP (OR=2.150) and BMI and MDA (OR=1.105). Adjusted R^2^ for the Model was 0.763, which means that even 76.3% of the variation in BMI could be explained with this Model. AOPP was not tested in multivariable analysis because its concentrations were not significantly associated with BMI in univariate analysis.

**Table 4:** Binary logistic regression analysis for the associations of biochemical data and BMI in adolescent girls.

Predictors	Unadjusted OR (95% CI)	P	R^2^
LDL-c, mmol/L	3.094 (0.935–10.235)	0.064	0.110
hsCRP, mg/L	2.495 (1.253–4.967)	0.009	0.347
Uric acid, μmol/L	1.024 (1.008–1.040)	0.009	0.190
MDA, μmol/L	1.062 (1.024–1.102)	0.001	0.467
AOPP, T/L	1.067 (1.010–1.127)	0.134	0.072
Model	Adjusted<br>OR (95% CI)	*P*	R^2^
LDL-c, mmol/L	11.766 (0.882–157.050)	0.062	0.763
hsCRP, mg/L	2.150 (1.139–4.057)	0.018	
Uric acid, μmol/L	1.004 (0.978–1.032)	0.754	
MDA, μmol/L	1.105 (1.028–1.187)	0.004	

## Discussion

This is the first study that investigated a broad spectrum of oxidative stress biomarkers in adolescent girls of a narrow age range (i.e. between 16 and 19 years old). Namely, previous studies included a wide age range of participants and the majority of them included both, children and adolescents. In order to elimi nate hormonal changes during puberty, as well as to minimize the other bias factors that might influence oxidative stress and inflammation, such as co-morbi dities, we have included only normal weight and overweight/obese girls who have a regular menstrual cycle.

The results of the current study showed that there was no difference in routine biochemical parameters, such as fasting glucose, creatinine and lipid status (except for HDL-c) between normal weight and overweight/obese girls. However, overweight/obese girls exhibited higher inflammation (i.e., hsCRP) and higher oxidative stress level (i.e., uric acid, MDA and NOx). Moreover, hsCRP and MDA independently correlated with BMI.

We have previously reported that hsCRP correlated with surrogate markers of insulin resistance [i.e., HOMA-IR [22] and HDL-c/TG ratio [23]] and cardiovascular risk [9] which is in line with the findings of higher inflammation in obesity-related cardiometabolic disorders [8] [16] [24] [25].

Although many previous studies examined the influence of obesity and obesity-related disorders on oxidative stress, no universal biomarker was established. Also, when MDA is concerned discrepant results were reported between studies. Higher serum MDA levels were shown in some studies [26] [27] [28] [29] [30], whereas no difference between study groups was found in the others [31] [32] [33] [34] [35] [36].

Possible explanations for these discrepancies may be attributed to the different sample sizes, variations in age of the examined participants, comorbidities, as well as variations in duration and the extent of obesity.

The small sample size is one of the limitations of this study. However, previous studies also included a relatively small number of participants [26] [35] [36]. Aztatzi-Aguilar et al. [36] included an even smaller number of participants, i.e., 35 students with a median age of 16 years. Of them, 12 were normal weight and 23 were overweight/obese. Unlike our findings, they reported neither difference in MDA, nor in uric acid levels. Mizgier et al. [35] included a total of 37 normal weight and 22 overweight/obese girls with PCOS. Neither did they find the difference in MDA nor in CRP. A total of 62 children were encompassed (of the 32 with obesity) in a study of Dokumacioglu et al. [26] Serum MDA values were higher than those of the control group which is consistent with our results. The study of Zalewska et al. [27] included 40 normal weight (of them 20 teenagers), 20 overweight (of them 10 teenagers) and 20 obese (of them 10 teenagers) adolescents aged 11-18 years and showed higher MDA and uric acid in overweight/obese participants compared to normal weight counterparts.

Also, discrepant results in antioxidant enzyme activity was reported. Low SOD, higher GPx, but no difference in CAT activity and MDA was shown in over weight (n=36) and obese (n=33) adult participants, compared with normal weight (n=23) counter parts. However, visceral abdominal fat positively correlated with lipoperoxides (i.e., MDA is its main product) [31].

Similarly to our results, Monserrat-Mesquida et al. [28] showed higher MDA, and no difference in CAT in women with metabolic syndrome (n= 40), compared to those without metabolic syndrome (n= 40). Adenan et al. [33] examined a total of 80 female adults [normal weight (n= 23), overweight (n= 28) and obese (n= 29)], and found a higher CAT activity in the females with obesity compared with normal weight women, although no differences in MDA levels were shown. They explained such results by increased removal of ROS by enhanced CAT activity or the possibility that MDA increase in the obese group was at low level that cannot be determined by the assays. A positive correlation was observed between MDA and BMI in women with PCOS [30].

Similarly, an animal experimental study that included a total of 28 Wistar-Bratislava white male rats showed that dyslipidemia, hypertension, and diabetes mellitus were associated with an increase in serum MDA levels [37]. Another experimental study [38] showed that isolated adipocytes from adipose tissue from mice fed on a diet enriched with fat were characterized by insulin resistance and a twofold increase in the production of ROS which led to metabolic syndrome. Moreover, obese mice with type 2 diabetes mellitus exhibited increase in MDA levels in plasma and white adipose tissue, and lower antioxidant enzyme activity as compared with nonmetabolic syndrome mice [39].

Oxidative stress can lead to reduced activity of phosphatidylinositol 3-kinase (PI3K). The letter represents the key enzyme responsible for insulindependent signaling and takes part in the ROSinduced insulin resistance formation. At the same time, the activity of protein kinase Cδ (PKC-δ) and the activity of janus kinase (JAK) in adipocytes are increased. All these pathophysiological processes may contibute to obesity-related metabolic disorders [40].

As previously stated, we have also found higher serum NOx levels in overweight/obese girls, as compared to normal weight peers. This is contrary to our previous finding where there was no difference in this biomarker in the adult population with and without metabolic syndrome. This may be attributed to confounding factors, such as medication use, smoking habits and other co-morbidities in the latter study [16]. Stable end metabolites, i.e., inorganic nitrates and nitrites (NOx) represent the NO production as one of the biomarkers of nitro-oxidative stress and the key indicator of energy metabolism and vascular tone [12]. It is synthesized from L-arginine under the control of NO synthase (NOS), an enzyme that is presented in 3 isoforms (i.e., inducible, neuronal and endothelial) with inducible NOS with the best capacity for NO formation in the state of oxidative stress [12] [16].

On the contrary, we found no difference in AOPP between examined groups, although positive correlation between BMI and AOPP in the whole group of participants was shown. AOPP is a biomarker that reflects the proteins' oxidative damage and was reported to be higher in some other metabolic disorders [8] [16] [41].

We also did not observe any difference between XOD, XO and XDH. XO takes part in the differentiation of adipocytes by controlling the activity of the nuclear receptor peroxisome proliferator-activated receptor (PPAR) [42]. Although previous studies reported higher XO activity in obesity, these included a smaller and younger group of participants than we did [43] [44]. Tam et al. [43] evaluated 22 normal weight and 20 obese children and adolescents (mean age, 12±3 years) and showed a 3.8-fold increase in plasma XO activity in obese, compared to normal weight counterparts. Similarly, Chiney et al. [44] included even younger participants, i.e., 9 obese prepubertal children and 16 normal weight between ages 6-10 years. However, in our study that included 118 overweight/obese adults, we have confirmed the association between XO and BMI [14].

Besides the small sample-size as mentioned above, this study cannot confirm causality due to its cross-sectional design. On the other hand, the strength of the current study lies in the fact that we included only late-adolescent girls who were nonsmokers, without co-morbidities and without any medication use. Thus, we put an effort into minimizing bias factors that might affect oxidative stress level. Moreover, we investigated a broad spectrum of nitro-oxidative stress biomarkers to gain deeper insight into the relationship between patho physiolo gical traits of obesity and nitro-oxidative stress.

## Conclusion

The increased level of nitro-oxidative stress was observed in late-adolescent girls. Although not all biomarkers of oxidative stress differed between normal weight and overweight/obese girls (i.e., AOPP, XOD, XDH, XO), neither difference in CAT activity between those groups was shown, the hsCRP and MDA independently correlated with BMI. Since obesity in adolescents often tracks into the adult period, leading to many obesity-related disorders, more studies with larger sample size and with longitudinal design are needed to confirm the causal link between obesity and oxidative stress and to find the best therapeutic approach to this issue.

## Dodatak

### Acknowledgement

This research was partially funded by a grantfrom the Ministry of Science, Montenegro and the Ministry of Education, Science and Technological Development, Republic of Serbia through Grant Agreement with University of Belgrade-Faculty of Pharmacy No: 451-03-68/2022-14/200161.

### Conflict of interest statement

All the authors declare that they have no conflictof interest in this work.
